# Resveratrol Alleviates Vascular Endothelial Damage Caused by Lower-Extremity Ischemia Reperfusion (I/R) through Regulating Keap1/Nrf2 Signaling-Mediated Oxidative Stress

**DOI:** 10.1155/2021/5556603

**Published:** 2021-03-24

**Authors:** Xiaojun Song, Zhili Liu, Rong Zeng, Jiang Shao, Yuehong Zheng, Wei Ye

**Affiliations:** Department of Vascular Surgery, Peking Union Medical College Hospital, Beijing 100730, China

## Abstract

The present study aims to investigate the protective effects of Resveratrol (RSV) against vascular endothelial damage caused by lower-extremity I/R and the underlying preliminary mechanism. The in vitro hypoxia reoxygenation (HR) model was established on HUVECs. Lower-extremity I/R model was established on rats followed by being treated with RSV and the pathological state of artery was evaluated by HE and EVG staining, while the apoptotic state of artery was detected by TUNEL assay. The cell viability was detected by MTT assay and the apoptotic state of cells was determined by Hoechst test and flow cytometry assay. DCFH-DA staining was used to measure the level of ROS and the production of MDA and SOD was measured by commercial kits. The expression level of Nrf2, Keap1, HO-1, Bcl-2, Bax, and Caspase-3 in cells was determined by Western blot. Nrf2 was knocked down by siRNA technology. Overall, our data indicated that increased cell viability, declined apoptotic rate, and alleviated oxidative stress were observed in RSV treated HR HUVECs, which were significantly reversed by knocking down Nrf2. Animal experiment revealed that the pathological and apoptotic state of femoral artery were dramatically ameliorated by the treatment of RSV, accompanied by the alleviated oxidative stress, which were abolished by the co-administration of ML385, an inhibitor of Nrf2. Taken together, our data revealed that RSV might alleviate vascular endothelial injury induced by lower-extremity I/R injury through regulating Keap1/Nrf2 signaling-mediated oxidative stress.

## 1. Introduction

Acute lower extremities ischemia mainly results from interrupted flow of the distal artery in the limb induced by arterial thrombosis, arterial injury, and arterial emboli of lower limbs, which is a common arterial disease in vascular surgery department [1]. Myocyte necrosis, as well as the loss of sensation and movement, will be observed approximately 3 hours post-ischemia, with irreversible injury induced about 6 hours later [2]. Due to high disability rate and mortality, public health and safety are being severely threatened by acute lower extremities ischemia [3]. The normal metabolism and function of tissues are significantly impacted by the prolonged tissue or organ ischemia and restoring blood supply to ischemic tissues and organs is an indispensable condition to recover normal functions. However, further damage will be induced to tissues by restoring blood supply following prolonged ischemia, which is defined as lower-extremity ischemia reperfusion (I/R) injury [4]. Lower-extremity I/R damage is mainly pathologically characterized as cell swelling, inflammatory cell infiltration, and cell apoptosis, which further contribute to systemic inflammation, damaged respiratory, renal, and hepatic function [5]. It is reported that series of inflammatory processes are involved in the development of lower-extremity I/R injury, including infiltration of neutrophile granulocytes, injured endothelial cells, excessive released cytokines, and superabundant production of reactive oxygen species (ROS) [6], among which ROS, as well as the induced oxidative stress, plays an important role as the initiating step of oxidative stress [7]. ROS is mainly generated from neutrophile granulocytes, myocytes, vascular endothelial cells, and perivascular tissues. Under normal circumstances, the production and elimination of ROS are maintained as an equilibrium state. However, once the excessive produced ROS is not cleared, oxidative stress state will be induced, which contributes to the peroxidation of intracellular components in multiple tissues, oxidative damage, and finally tissue dysfunctions [8]. When ischemia occurs, aerobic respiration will be suppressed and anaerobic respiration will be facilitated due to hypoxia, which result in weakened oxidative reaction and increased release of lactic acid. However, as the reperfusion of blood flow, a great deal of oxygens will be instantly released into the body, which contributes to the excessive production of ROS. As a consequence, the cellular components will be oxidized and tissue injury will be induced by series of inflammatory reactions [9]. Therefore, oxidative stress might be an important target for the treatment of lower-extremity I/R injury.

Resveratrol (RSV) is a natural polyacid rich in red wine and has been proved to be healthful [10]. More and more bodies of evidence have claimed that the biofunction of RSV is closely related to its anti-oxidative stress property [11]. Rio reported that oxidative stress was significantly ameliorated in dystrophin-deficient *m*d*x* mice by RSV through alleviating mitophagy [12]. Huang reported that oxidative stress and mitochondrial dysfunction were dramatically prevented by RSV through regulating the PKA/LKB1/AMPK signaling [13]. This study aimed to investigate the protective function of RSV against in vitro hypoxia reoxygenation model and in vivo ischemia reperfusion model to explore the potential therapeutic property of RSV against lower-extremity I/R injury.

## 2. Materials and Methods

### 2.1. Cells and Hypoxia Reoxygenation (HR) Treatment

In the present study, for in vitro experiments, human umbilical vein endothelial cells (HUVECs) were obtained from BioVector NTCC (Beijing, China) and cultured in DMEM medium (Gibco, California, USA) containing 10% fetal bovine serum (FBS) and penicillin-streptomycin. The cultural condition for HUVECs was 5% CO_2_ and 37°C. To establish the in vitro HR model, the cells were incubated under the condition of 1% O_2_, 5% CO_2_, and 94% N_2_ for 2 hours, followed by incubation under normal condition (5%CO_2_ and 94% N_2_). Resveratrol and other chemicals were obtained from Sigma-Aldrich (California, USA).

### 2.2. Animals and the Establishment of Lower-Extremity I/R Injury Model and Design of Animal Experiments

The establishment of lower-extremity I/R injury model on rats was conducted according to the instruction described previously [14]. Forty-eight Sprague Dawley (SD) rats were obtained from Kay Biological Technology Co., Ltd. (Shanghai, China) and adapted for 7 days after arrival. The animals were anesthetized by intraperitoneal injection of 1% pentobarbital sodium and were fixed on the operation table. A 2 cm incision was opened on the root position of the right hind limb of rats, followed by ligating the superficial arteries and veins of the abdominal wall. Subsequently, the femoral sheath was opened and the femoral artery was separated, followed by carefully closing the proximal end of the femoral artery with a vascular clamp near the inguinal ligament. Then, the blood circulation of the collateral was blocked by a limb ring under the femoral sheath with a rubber band, followed by removing the clamp and rubber band 6 hours after the femoral artery was blocked. The state of blood flow was observed until the pulse and blood flow of rats returned to normal, followed by sewing up the incisions immediately. After the different treatment strategies, the animals were executed with euthanasia and the femoral artery tissues were collected. The incision was also opened on the animals in Sham group followed by sewing up the incisions immediately. The animals in I/R group were administered with normal saline orally for an 8-week consecutive dosing. The rats in RSV + I/R were dosed orally with 1 mg/kg/day RSV for an 8-week consecutive dosing [15, 16]. And the animals in RSV + I/R + ML385 group were administered orally with 1 mg/kg/day RSV combined with 30 mg/kg/week ML385 [17] for 8 weeks.

### 2.3. MTT Assay

The treated HUVECs were planted on 96-well plates as 2 × 10^4^ cells/well, followed by being added with 5 mg/mL MTT solution (MedChemExpress, New Jersey, USA) for 3-4 hours. Subsequently, approximately 150 *µ*L DMSO solution was added to each well to terminate the reaction. Finally, the optical density (OD) values were detected using the microplate reader (Thermo Fisher, MA, USA).

### 2.4. Hoechst Assay

Clean coverslips were put in 6-well plates and the cells were planted. Following different treating strategies, the medium was removed and approximately 500 *µ*L polyformaldehyde fixing solution was added to fix the cells for over 10 min. After blotting up the fixing solution, the cells were washed by PBS on decolorization shaker twice and about 500 *µ*L Hoechst solution was added for staining 5 min, followed by being washed twice. Finally, DAPI dyes were added to sealing and the pictures were taken under the fluorescence microscope (Thermo Fisher, MA, USA).

### 2.5. Flow Cytometry Assay

The treated HUVECs were collected and adjusted in the centrifuge tube at a density of 2 × 10^6^/mL, followed by centrifugation at 4°C and 1000 r/min for 10 min. Subsequently, approximately 200 *µ*L binding buffer was instilled and 5 *µ*L Annexin V-FITC and PI solution were instilled, respectively. After being incubated for 15 min, the samples were loaded in the flow cytometry (BD, New York, USA) for detection.

### 2.6. Detection of ROS Level

Levels of ROS in treated HUVECs were evaluated by DCFH-DA fluorescence probe assay. In brief, the cells were planted on 6-well plates, followed by different treating strategies. Subsequently, 10 *µ*mol/L DCFA-DA fluorescence probe was added to be further incubated for 30 min. After being washed using serum-free DMEM medium, the images were taken under the inverted microscope (Olympus, Tokyo, Japan).

### 2.7. MDA and SOD Detection

After the cells were planted on 24-well plates, treated HUVECs following different treating strategies were crushed by ultrasound, followed by collecting the supernatant. The concentration of MDA and SOD in the samples was detected utilizing the commercial kit according to the instruction of the manufacturers.

### 2.8. Western Blot Assay

The treated HUVECs and the isolated vascular were collected and added with lysis buffer (PMDF : RIPA = 1 : 99) to isolate the total proteins, which were further quantified utilizing the BCA kit (Thermo fisher, MA, USA). Subsequently, the samples were loaded and separated by the sodium dodecyl sulfate polyacrylamide gel electrophoresis (SDS-PAGE), which were further transferred to PVEF membrane (Thermo Fisher, MA, USA) and incubated with 5% skim milk to block the non-specific binding proteins. Then, the membrane was incubated with primary antibodies against Nrf2 (1 : 1000, Santa Cruz Biotechnology, California, USA), Keap1 (1 : 1000, Santa Cruz Biotechnology, California, USA), HO-1 (1 : 1000, Santa Cruz Biotechnology, California, USA), Bcl-2 (1 : 1000, Santa Cruz Biotechnology, California, USA), Bax (1 : 1000, Santa Cruz Biotechnology, California, USA), Caspase-3 (1 : 1000, Santa Cruz Biotechnology, California, USA), or GAPDH (1 : 1000, Santa Cruz Biotechnology, California, USA) at 4°C overnight, followed by being incubated with secondary antibody (1 : 2000, CST, Boston, USA) at room temperature for 2 hours. Lastly, the membrane was added with ECL solution and exposed to Tanon 4600 (Tanon, Shanghai, China). Images were analyzed by Image *J* software.

### 2.9. Transfection

The Nrf2 knockout (Nrf2 KO) HUVECs were established by transfecting the siRNA targeting Nrf2. Briefly, the cells were planted on 6-well plates and were transfected with siRNAs along with the transfection reagents (Lipofectamine 3000, Thermo Fisher, MA, USA), which were further incubated for 24 hours. The efficacy of transfection was confirmed by Western blot assay. The sequence for siRNA targeting Nrf2 was listed as follows: forward: 5′-GGCGCCTAATTGTCAACTTCTG-3′; reverse: 5′-GTGCAGGGTCCGAGGT-3′.

### 2.10. TUNEL Assay

The isolated femoral artery tissues were fixed in 4% paraformaldehyde solution for 6 hours and embedded by paraffin, followed by being stored at 4°C in 30% sucrose solution for 3 days. Subsequently, the tissues were sectioned (18 *µ*m) and washed by PBS buffer, followed by being added with TUNEL reaction solution at 37°C for 1 hour in the dark. After washing the slides with PBS 3 times, DAB reagent was added for chromogenic experiment, followed by taking images with optical microscope (Olympus, Tokyo, Japan) to evaluate the apoptotic state of femoral artery tissues.

### 2.11. HE and EVG Staining

The slides were soaked in xylene solution twice, followed by soaking in 100%, 95%, 85%, and 70% ethanol solution successively.

For HE staining, after washing with PBS buffer several times, the slides were stained with Hematoxylin dye for 3–5 min, followed by incubating in acid for 40 s and in ammonia solution for 40 s. Subsequently, the slides were stained in eosin dye for 2 min, followed by soaking in xylene solution for 5 min. Finally, the sections were sealed with neutral gum and observed under the inverted microscope (Olympus, Tokyo, Japan).

For EVG staining, the slides were stained with EVG solution (Hematoxylin, iodine solution, and ferric chloride mixed at a ratio of 5 : 2:2), followed by incubation for 30 min and washed with water. The background was differentiated by ferric chloride differentiation solution. The above steps were repeated until the background was grey white. Subsequently, the slides were re-dyed with VG solution (saturated picric acid and fuchsin solution mixed at a ratio of 9 : 1), followed by being washed several times and quick dehydration using 100% ethanol. Finally, the sections were sealed with neutral gum and observed under the inverted microscope (Olympus, Tokyo, Japan).

### 2.12. Statistical Analysis

The data obtained in the present study was analyzed using the SPSS 22.0 software and plotted utilizing GraphPad Prism 8. The data was presented as mean ± SD. The data between two groups was compared with *t*-test and the data among multiple groups was compared with one-way ANOVA analysis. *p* < 0.05 was regarded as significant in the present study.

## 3. Results

### 3.1. RSV Protected HUVECs from Injury Induced by HR

**Figure 1:**
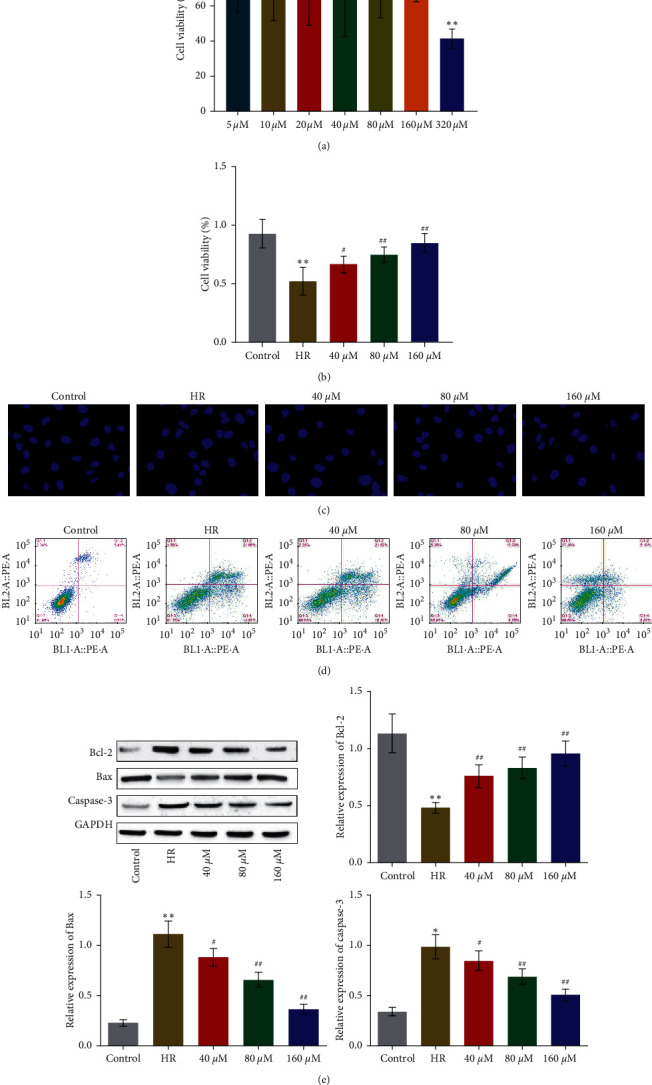
The HR-induced injury on HUVECs was alleviated by RSV. (a) The cell viability of HUVECs treated with different concentration of RSV was evaluated by MTT assay (^*∗∗*^*p* < 0.01 vs. 5 *µ*M). (b) The cell viability of HUVECs treated with different strategies was detected by MTT assay (^*∗∗*^*p* < 0.01 vs. control, #*p* < 0.05 vs. HR, and ##*p* < 0.01 vs. HR). (c) The apoptotic state of HUVECs treated with different strategies was measured by the Hoechst test. (d) The apoptotic rate of HUVECs treated with different strategies was determined by flow cytometry. (e) The expression level of Bcl-2, Bax, and Caspase-3 was detected by Western blot (^*∗∗*^*p* < 0.01 vs. control, #*p* < 0.05 vs. HR, and ##*p* < 0.01 vs. HR).

Firstly, we explored the highest tolerant concentration for RSV to be incubated in HUVECs. As shown in Figure 1(a), no significant difference was observed on cell viability as the concentration of RSV was increased from 5 *µ*M to 160 *µ*M. However, as the concentration of RSV increased from 160 *µ*M to 320 *µ*M, the cell viability was dramatically suppressed (^*∗∗*^*p* < 0.01 vs. 5 *µ*M). To further investigate the protective effect of RSV against HR treated HUVECs, cells were cultured under HR condition in the absence or presence of RSV (40, 80, and 160 *µ*M) for 24 hours. As shown in Figure 1(b), the cell viability was significantly inhibited by HR culture, which was greatly elevated by the introduction of RSV (^*∗∗*^*p* < 0.01 vs. control, #*p* < 0.05 vs. HR, ##*p* < 0.01 vs. HR). We further investigated the state of apoptosis of treated HUVECs utilizing Hoechst assay and flow cytometry. As shown in Figure 1(c), compared to control, round nucleus and accumulated chromatin were observed in HR treated HUVECs, which were significantly alleviated by the treatment of RSV, indicating an obviously inhibitory effect of RSV against apoptosis induced by HR. In addition, compared to control, the apoptotic rate (Figure 1(d)) was increased from 6.01% to 36.65% in HR treated HUVECs, which was suppressed to 28.64% and 14.01% by the introduction of 80 and 160 *µ*M RSV, respectively. Lastly, we found that the expression of Bcl-2 was significantly inhibited and the expression of Bax and Caspase-3 was dramatically elevated induced by HR treatment, which were greatly reversed by the incubation of RSV (^*∗∗*^*p* < 0.01 vs. control, #*p* < 0.05 vs. HR, ##*p* < 0.01 vs. HR).

### 3.2. RSV Alleviated Oxidative Stress in HUVECs Induced by HR

**Figure 2:**
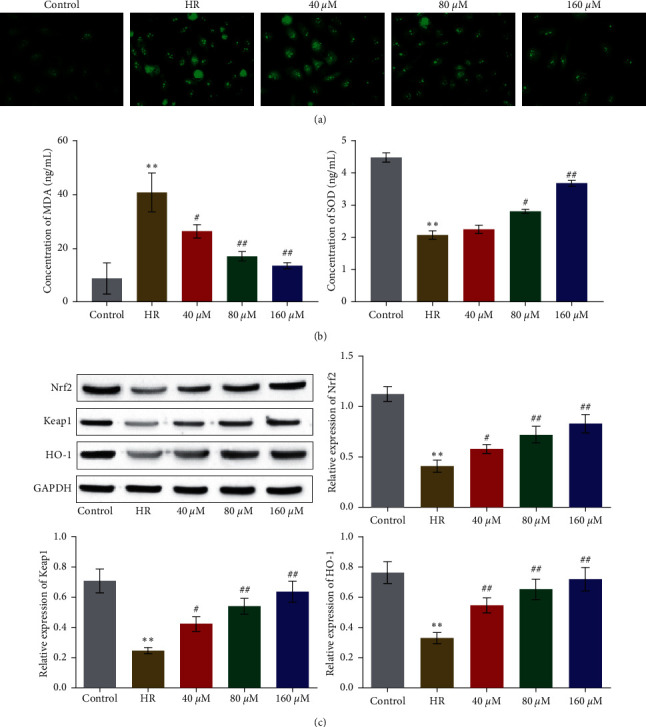
HR-induced oxidative stress on HUVECs was ameliorated by RSV. (a) The ROS level in HUVECs treated with different strategies was measured by DCFH-DA assay. (b) The concentration of MDA and SOD was determined by commercial kits. (c) The expression of Nrf2, Keap1, and HO-1 was evaluated by Western blot (^*∗∗*^*p* < 0.01 vs. control, #*p* < 0.05 vs. HR, and ##*p* < 0.01 vs. HR).

To evaluate the state of oxidative stress in HUVECs, the level of ROS, MDA, and SOD was detected. As shown in Figure 2(a), the fluorescence intensity of DCFA-DA was significantly increased in HR treated HUVECs, which was greatly declined by the introduction of different dosage of RSV. Compared to control, the level of MDA (Figure 2(b)) was increased from 8.7 ng/mL to 40.9 ng/mL by the treatment of HR, which was suppressed to 26.3, 17.0, and 13.5 ng/mL by the introduction of 40, 80, and 160 *µ*M RSV, respectively (^*∗∗*^*p* < 0.01 vs. control, #*p* < 0.05 vs. HR, ##*p* < 0.01 vs. HR). In addition, compared to control, the concentration of SOD was significantly inhibited from 4.5 ng/mL to 2.1 ng/mL in HR treated HUVECs, which was elevated to 2.8 and 3.7 ng/mL by the administration of 80 and 160 *µ*M RSV, respectively (^*∗∗*^*p* < 0.01 vs. control, #*p* < 0.05 vs. HR, ##*p* < 0.01 vs. HR). To further investigate the underlying mechanism, the impact of RSV on Nrf2 signaling was evaluated. As shown in Figure 2(c), the expression of Nrf2, Keap1, and HO-1 was significantly suppressed by the treatment of HR, which was greatly elevated by the introduction of RSV (^*∗∗*^*p* < 0.01 vs. control, #*p* < 0.05 vs. HR, ##*p* < 0.01 vs. HR). These data indicated that oxidative stress induced by HR was dramatically ameliorated by RSV.

### 3.3. The Protective Effect of RSV against HR-Treated HUVECs Was Abolished by the Knockout of Nrf2

**Figure 3:**
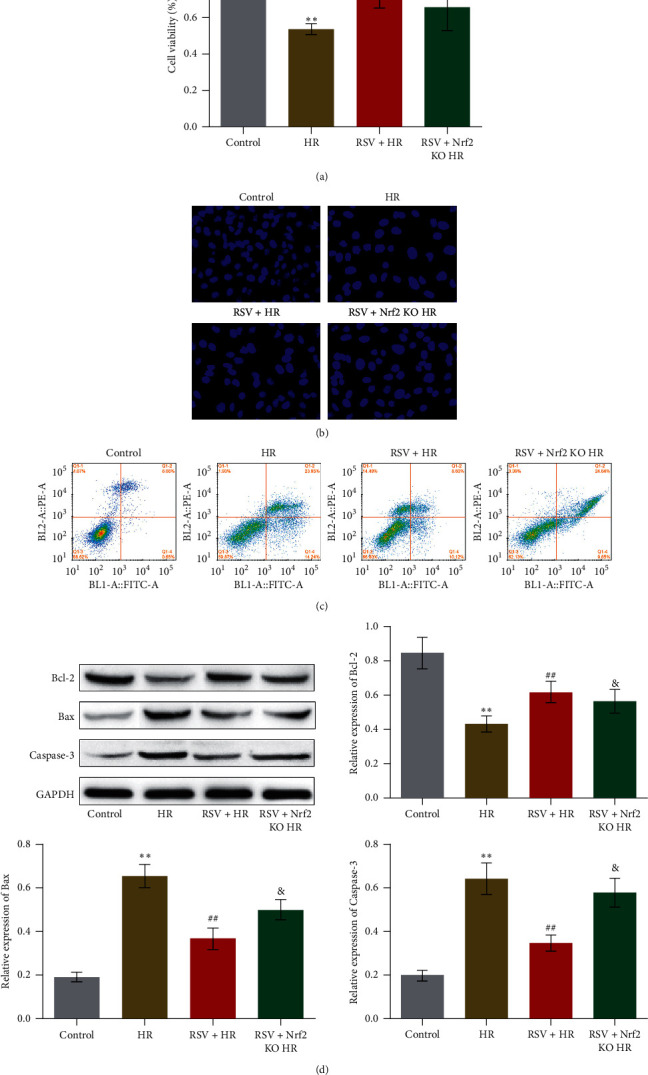
The protective effect of RSV against HR-treated HUVECs was abolished by knocking down Nrf2. (a) The cell viability of HUVECs treated with different strategies was detected by MTT assay. (b) The apoptotic state of HUVECs treated with different strategies was measured by Hoechst test. (c) The apoptotic rate of HUVECs treated with different strategies was determined by flow cytometry. (d) The expression level of Bcl-2, Bax, and Caspase-3 was detected by Western blot (^*∗∗*^*p* < 0.01 vs. control, ##*p* < 0.01 vs. HR, and &*p* < 0.05 vs. RSV + HR).

To further verify the involvement of Nrf2 signaling in the protective effect of RSV against HR-treated HUVECs, the HR incubated Nrf2 KO HUVECs were established and treated with RSV (RSV + Nrf2 KO HR). As shown in Figure 3(a), the declined cell viability in HR-treated HUVECs was significantly elevated by the introduction of RSV, which was further suppressed by the knocking down of Nrf2 (^*∗∗*^*p* < 0.01 vs. control, ##*p* < 0.01 vs. HR, &*p* < 0.05 vs. RSV + HR). We further detected the apoptotic state of HUVECs from different groups. As shown in Figure 3(b), compared to RSV + HR group, round nucleus and accumulated chromatin were observed in RSV + Nrf2 KO HR group. In addition, compared to RSV + HR group, the apoptotic rate of HUVECs was increased from 18.72% to 34.49% in the RSV + Nrf2 KO HR group (Figure 3(c)). Lastly, as shown in Figure 3(d), compared to RSV + HR group, Bcl-2 was significantly downregulated and Bax and Caspase-3 were dramatically upregulated in RSV + Nrf2 KO HR group (&*p* < 0.05 vs. RSV + HR). These data indicate that the protective effect of RSV against HR-treated HUVECs was abolished in Nrf2 KO HUVECs.

### 3.4. The Protective Effect of RSV against HR-Induced Oxidative Stress Was Abolished by the Knockout of Nrf2

**Figure 4:**
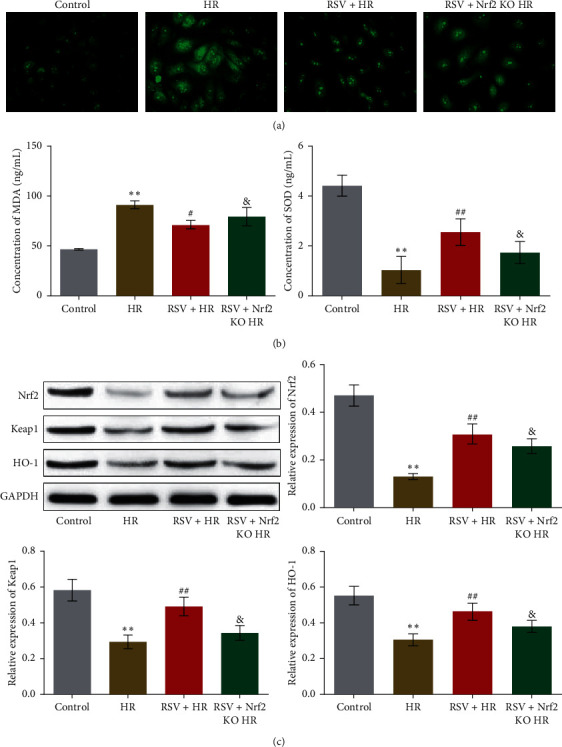
The protective effect of RSV against oxidative stress in HUVECs induced by HR was abolished by knocking down Nrf2. (a) The ROS level in HUVECs treated with different strategies was measured by DCFH-DA assay. (b) The concentration of MDA and SOD was determined by commercial kits. (c) The expression of Nrf2, Keap1, and HO-1 was evaluated by Western blot (^*∗∗*^*p* < 0.01 vs. control, #*p* < 0.05 vs. HR, ##*p* < 0.01 vs. HR, and &*p* < 0.05 vs. RSV + HR).

We further detected the state of oxidative stress in HUVECs treated with different strategies. As shown in Figure 4(a), decreased fluorescence intensity in RSV + HR group was significantly elevated in RSV + Nrf2 KO HR group (&*p* < 0.05 vs. RSV + HR), indicating that the suppressed production of ROS induced by RSV was greatly reversed by knocking down the expression of Nrf2. In addition, compared to RSV + HR group, the concentration of MDA (Figure 4(b)) was increased from 71.48 ng/mL to 79.57 ng/mL and the concentration of SOD was decreased from 2.56 ng/mL to 1.74 ng/mL in RSV + Nrf2 KO HR group (&*p* < 0.05 vs. RSV + HR). As for the state of Nrf2 signaling (Figure 4(c)), compared to RSV + HR group, the expression of Nrf2, Keap1, and HO-1 was dramatically inhibited in RSV + Nrf2 KO HR group (&*p* < 0.05 vs. RSV + HR). These data indicated that knocking down Nrf2 abolished the protective effect of RSV against HR-induced oxidative stress.

### 3.5. RSV Ameliorated the Pathological Symptom in Lower-Extremity I/R Rats

**Figure 5:**
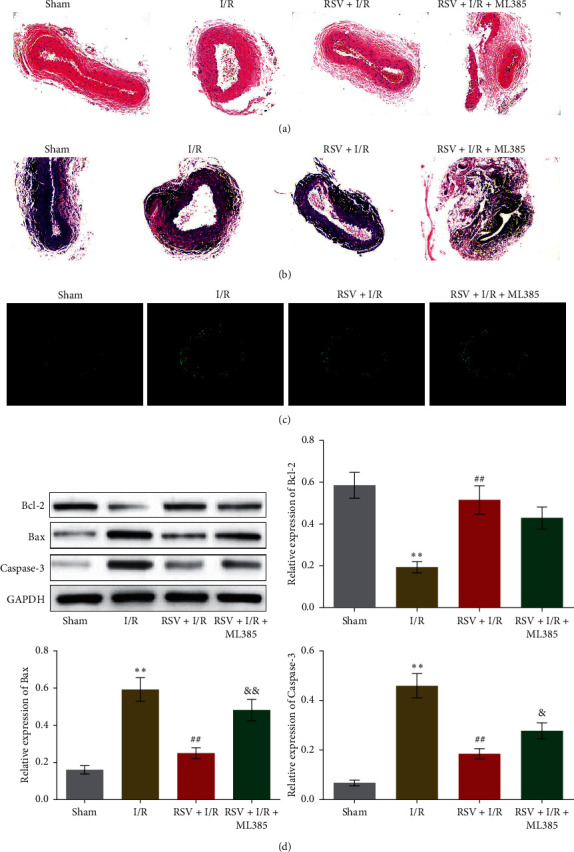
RSV alleviated the pathological changes in femoral artery tissues from low-extremity I/R rats. (a, b) The pathological state of femoral artery tissues was evaluated by HE and EVG staining. (c) The apoptotic state of femoral artery tissues was measured by TUNEL staining assay. (d) The expression level of Bcl-2, Bax, and Caspase-3 was detected by Western blot (^*∗∗*^*p* < 0.01 vs. Sham, ##*p* < 0.01 vs. I/R, &*p* < 0.05 vs. RSV + I/R, and &&*p* < 0.01 vs. RSV + I/R).

To explore the potential therapeutic effect of RSV against lower-extremity I/R, the lower-extremity I/R model was established followed by RSV treatments. As shown in Figures 5(a) and 5(b), in Sham group, the boundary among intima, media, and adventitia was clear. The structure of these membranes was integrated. Monolayer of endothelial cells in alignment was observed in the intima, connected by tight junctions. Collagen fiber and elastic fiber were abundant, and the structure was clear without fracture in media membrane. The adventitia was composed of connective tissues and integrated with no significant thickening, fracture, or defect. In I/R group, indistinct boundary and incrassation were observed in intima, media, and adventitia. Increased volume of endothelial cells, rough lumen surface, and small amount of platelet adhesion were observed in intima. Disordered thickening, degeneration and fracture of fiberboard, and infiltration of inflammatory cells were observed in media membrane. Fiber connective tissues characterized with edema, thickening, local fracture, and defect were observed in adventitia. In RSV + I/R group, the degree of endothelium edema, thickening vascular wall, infiltration of inflammatory cells, and damaged elastic fiber layer were obviously alleviated, which, however, were not observed in RSV + I/R + ML385 group. ML385 is a promising Nrf2 inhibitor widely used in experimental studies. We further investigated the apoptotic state in femoral artery tissues. As shown in Figure 5(c), compared to Sham group, the increased number of apoptosis bodies was observed in I/R group and was decreased in RSV + I/R group, which was greatly suppressed in RSV + I/R + ML385 group. In addition, the downregulated Bcl-2 and upregulated Bax and Caspase-3 in I/R group were significantly reversed by the treatment of RSV, which were further abolished by the coadministration of ML385 (^*∗∗*^*p* < 0.01 vs. Sham, ##*p* < 0.01 vs. I/R, &*p* < 0.05 vs. RSV + I/R). These data indicated that the pathological symptom in lower-extremity I/R rats was significantly ameliorated by RSV, which was further abolished by blocking Nrf2 signaling.

### 3.6. RSV Alleviated Oxidative Stress in the Femoral Artery Tissues of Lower-Extremity I/R Rats

**Figure 6:**
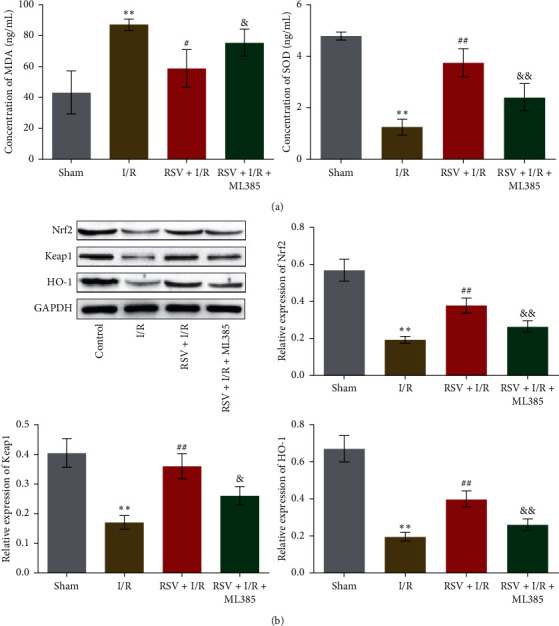
RSV alleviated oxidative stress in femoral artery tissues from low-extremity I/R rats. (a) The production of MDA and SOD in femoral artery tissues was detected by commercial kits. (b) The expression level of Nrf2, Keap1, and HO-1 was evaluated by Western blot (^*∗∗*^*p* < 0.01 vs. Sham, #*p* < 0.05 vs. I/R, ##*p* < 0.01 vs. I/R, &*p* < 0.05 vs. RSV + I/R, and &&*p* < 0.01 vs. RSV + I/R).

As shown in Figure 6(a), compared to Sham group, the concentration of MDA in femoral artery tissues was increased from 43.36 ng/mL to 87.27 ng/mL in I/R group, which was further decreased to 58.83 ng/mL in RSV + I/R group. In addition, the concentration of MDA in femoral artery tissues was elevated to 75.74 in RSV + I/R + ML385 group. Compared to Sham group, the concentration of SOD in femoral artery tissues was declined from 4.79 ng/mL to 1.26 ng/mL in I/R group and was promoted to 3.75 ng/mL in RSV + I/R group, which was further decreased to 2.43 ng/mL in RSV + I/R + ML385 group. As shown in Figure 6(b), the decreased expression of Nrf2, Keap1, and HO-1 in femoral artery tissues in I/R group was significantly elevated in RSV + I/R group, which was further suppressed in RSV + I/R + ML385 group (^*∗∗*^*p* < 0.01 vs. Sham, ##*p* < 0.01 vs. I/R, &*p* < 0.05 vs. RSV + I/R, &&*p* < 0.01 vs. RSV + I/R). These data indicated that oxidative stress in femoral artery tissues of lower-extremity I/R rats was significantly alleviated by the treatment of RSV, which was abolished by blocking Nrf2 signaling.

## 4. Discussion

Vascular endothelial cells are considered the first barrier for the protection of vessels, which play an important role in wound healing, thrombosis, and neovascularization [18]. It is widely reported that injures on vascular endothelial cells are involved in the pathological progress of lower-extremity I/R injury [19]. In the process of I/R injury, apoptosis of vascular endothelial cells is commonly observed. Wu et al. reported that apoptosis induced vascular endothelial cell injury was involved in the pathological process of lower-extremity I/R injury, which was closely related to AMPK signaling [20]. In addition, the upregulation of Bax and Caspase-3, proapoptotic factors [21], and the downregulation of Bcl-2 [22], an anti-apoptotic factor, in vascular endothelial cells are reported to be observed in I/R injury animal models [23]. In the present study, we used HR cultural condition to simulate the pathological state of HUVECs in lower-extremity I/R, which was verified by the decreased cell viability and elevated apoptotic rates. By the treatment of RSV, the cell viability and apoptotic rate were significantly alleviated, indicating a promising protective effect of RSV against endothelial injury induced by HR condition. Further verifications were conducted in lower-extremity I/R rats. Significant pathological changes, including endothelium edema, thickening vascular wall, infiltration of inflammatory cells, damaged elastic fiber layer, and increased number of apoptotic body, were observed in lower-extremity I/R rats, which were consistent with the description reported previously [24]. Following 8 weeks' consecutive treatment of RSV, the pathological changes were significantly reversed, indicating a pronounced protective property of RSV against lower-extremity I/R endothelial injury. In our further work, more indexes will be included to fully evaluate the therapeutic effect of RSV on lower-extremity I/R injury, including measuring the thickness of intima, media, and adventitia of arterial wall, as well as the measurement of arterial lumen area.

In ischemia diseases, the rapid onset, the duration, and severity of ischemia are regarded as the main elements responsible for the tissue's damages. As the blood flow is restored, abundant oxygens and nutrient substances get into the cells, which contribute to the rebooting of aerobic oxidation and the abnormal activation of tissue metabolism. Subsequently, the production of ROS was accelerated, which further results in the activation of nicotinamide adenine dinucleotide phosphate (NADPH), the excessive production of nitric oxide (NO), and the release of cytochrome C. As a consequence, the irreversible damage on tissues and cells will be aggravated [25]. Several mechanisms are reported on the injury induced by excessive released ROS and oxidative stress. Firstly, the permeability of cell membrane and lysosomal membrane can be changed by ROS, which induces transmembrane transport disorder. As a result, the proteases are activated, DNA dissolution is trigged, and the protein denaturation is induced, which finally activate the apoptotic signaling and the onset of apoptosis. Secondly, excessive release of inflammatory factors and chemotaxis of leukocytes will be mediated by ROS, which contribute to the development and processing the severe inflammatory reactions. Thirdly, the permeability of mitochondrial membrane will be changed by ROS, which subsequently blocks the respiratory chain, induces the metabolic disorder, and disrupts the cellular structure [26]. In the present study, oxidative stress was observed both in HR treated HUVECs and in femoral artery tissues isolated from lower-extremity I/R rats, which were consistent with the description reported previously [27, 28]. After 8 weeks' consecutive treatment of RSV, the state of oxidative stress both in HR treated HUVECs and in femoral artery tissues isolated from lower-extremity I/R rats was dramatically alleviated, indicating a promising protective effect of RSV against oxidative stress, which was consistent with the reports proposed by Zhuang et al. [29] and Sebori [12]. To further confirm that the protective effect of RSV against I/R injury was related to the inhibition of oxidative stress, we verified the effects of RSV by blocking the Nrf2 signaling, which is a well-known regulatory signal pathway against oxidative stress [30]. We found that the protective effect of RSV against both I/R injury and oxidative stress was significantly abolished, indicating that RSV might alleviate the I/R injury by ameliorating oxidative stress in endothelial cells. In our future work, the downstream impact of oxidative stress, such as excessive release of inflammatory factors and infiltration of inflammatory cells, will be investigated to further confirm the mechanism concluded in the present study.

## 5. Conclusion

Our data indicated that RSV might alleviate vascular endothelial injury induced by lower-extremity I/R injury through regulating Keap1/Nrf2 signaling-mediated oxidative stress.

## Data Availability

All the data in this study are included within the manuscript.

## References

[1] Bonicolini E., Martucci G., Simons J. (2019). Limb ischemia in peripheral veno-arterial extracorporeal membrane oxygenation: a narrative review of incidence, prevention, monitoring, and treatment. *Critical Care*.

[2] Zettervall S. L., Marshall A. P., Fleser P., Guzman R. J. (2018). Association of arterial calcification with chronic limb ischemia in patients with peripheral artery disease. *Journal of Vascular Surgery*.

[3] Katzen B. T. (2002). Clinical diagnosis and prognosis of acute limb ischemia. *Reviews in Cardiovascular Medicine*.

[4] Anzell A. R., Maizy R., Przyklenk K., Sanderson T. H. (2018). Mitochondrial quality control and disease: insights into ischemia-reperfusion injury. *Molecular Neurobiology*.

[5] Green T. R., Bennett S. R., Nelson V. M. (1994). Specificity and properties of propofol as an antioxidant free radical scavenger. *Toxicology and Applied Pharmacology*.

[6] Kan C., Ungelenk L., Lupp A., Dirsch O., Dahmen U. (2018). Ischemia-reperfusion injury in aged livers-the energy metabolism, inflammatory response, and autophagy. *Transplantation*.

[7] Guillot M., Charles A.-L., Chamaraux-Tran T. N. (2014). Oxidative stress precedes skeletal muscle mitochondrial dysfunction during experimental aortic cross-clamping but is not associated with early lung, heart, brain, liver, or kidney mitochondrial impairment. *Journal of Vascular Surgery*.

[8] Thiemermann C., Bowes J., Myint F. P., Vane J. R. (1997). Inhibition of the activity of poly (ADP ribose) synthetase reduces ischemia-reperfusion injury in the heart and skeletal muscle. *Proceedings of the National Academy of Sciences*.

[9] Mentzer R. M., Lasley R. D., Jessel A., Karmazyn M. (2003). Intracellular sodium hydrogen exchange inhibition and clinical myocardial protection. *The Annals of Thoracic Surgery*.

[10] Pervaiz S. (2003). Resveratrol: from grapevines to mammalian biology. *The FASEB Journal*.

[11] Shrikanta A., Kumar A., Govindaswamy V. (2015). Resveratrol content and antioxidant properties of underutilized fruits. *Journal of Food Science and Technology*.

[12] Sebori R., Kuno A., Hosoda R., Hayashi T., Horio Y. (2018). Resveratrol decreases oxidative stress by restoring mitophagy and improves the pathophysiology of dystrophin-deficient mdx mice. *Oxidative Medicine and Cellular Longevity*.

[13] Huang Y., Zhu X., Chen K. (2019). Resveratrol prevents sarcopenic obesity by reversing mitochondrial dysfunction and oxidative stress via the PKA/LKB1/AMPK pathway. *Aging*.

[14] Tatar T., Polat Y., Comu F. M., Kartal H., Arslan M., Kucuk A. (2018). Effect of cerium oxide on erythrocyte deformability in rat lower extremity ischemia reperfusion injury. *Bratislava Medical Journal*.

[15] Rocha K. K. R., Souza G. A., Ebaid G. X., Seiva F. R. F., Cataneo A. C., Novelli E. L. B. (2009). Resveratrol toxicity: effects on risk factors for atherosclerosis and hepatic oxidative stress in standard and high-fat diets. *Food and Chemical Toxicology*.

[16] Ferreira M. P., Willoughby D. (2008). Alcohol consumption: the good, the bad, and the indifferent. *Applied Physiology, Nutrition, and Metabolism*.

[17] Ling L., Tong J., Zeng L. (2020). Paeoniflorin improves acute lung injury in sepsis by activating Nrf2/Keap1 signaling pathway. *Sichuan Da Xue Xue Bao Yi Xue Ban*.

[18] Zhu T., Yao Q., Wang W., Yao H., Chao J. (2016). iNOS induces vascular endothelial cell migration and apoptosis via autophagy in ischemia/reperfusion injury. *Cellular Physiology and Biochemistry*.

[19] Shen G.-H., Song Y., Yao Y. (2020). Downregulation of DLGAP1-antisense RNA 1 alleviates vascular endothelial cell injury via activation of the phosphoinositide 3-kinase/Akt pathway results from an acute limb ischemia rat model. *European Journal of Vascular and Endovascular Surgery*.

[20] Wu Y., Zhang M. H., Xue Y. (2019). Effect of microRNA-26a on vascular endothelial cell injury caused by lower extremity ischemia-reperfusion injury through the AMPK pathway by targeting PFKFB3. *Journal of Cellular Physiology*.

[21] Erekat N. S., Stoker T. B., Greenland J. C. (2018). Apoptosis and its role in Parkinson’s disease. *Parkinson’s Disease: Pathogenesis and Clinical Aspects*.

[22] Tsujimoto Y. (1998). Role of Bcl-2 family proteins in apoptosis: apoptosomes or mitochondria?. *Genes to Cells*.

[23] Veres G., Hegedus P., Barnucz E. (2015). Endothelial dysfunction of bypass graft: direct comparison of in vitro and in vivo models of ischemia-reperfusion injury. *PLoS One*.

[24] Xu Y. L., Zhang M. H., Guo W. (2018). MicroRNA-19 restores vascular endothelial cell function in lower limb ischemia-reperfusion injury through the KLF10-dependent TGF-*β*1/Smad signaling pathway in rats. *Journal of Cellular Biochemistry*.

[25] McLaughlin B. A., Nelson D., Erecińska M., Chesselet M. F. (1998). Toxicity of dopamine to striatal neurons in vitro and potentiation of cell death by a mitochondrial inhibitor. *Journal of Neurochemistry*.

[26] Daenen K., Andries A., Mekahli D., Van Schepdael A., Jouret F., Bammens B. (2019). Oxidative stress in chronic kidney disease. *Pediatric Nephrology*.

[27] Hsu K.-Y., Tsai P.-S., Lee J.-J., Wang T.-Y., Huang C.-J. (2011). Platonin mitigates acute lung injury induced by bilateral lower limb ischemia-reperfusion in rats. *Journal of Surgical Research*.

[28] Zhang Q., Shang M., Zhang M. (2016). Microvesicles derived from hypoxia/reoxygenation-treated human umbilical vein endothelial cells promote apoptosis and oxidative stress in H9c2 cardiomyocytes. *BMC Molecular and Cell Biology*.

[29] Zhuang Y., Wu H., Wang X., He J., He S., Yin Y. (2019). Resveratrol attenuates oxidative stress-induced intestinal barrier injury through PI3K/Akt-Mediated Nrf2 signaling pathway. *Oxidative Medicine and Cellular Longevity*.

[30] Bellezza I., Giambanco I., Minelli A., Donato R. (2018). Nrf2-Keap1 signaling in oxidative and reductive stress. *Biochimica et Biophysica Acta (BBA)-Molecular Cell Research*.

